# Sexual Selection Comes at a Cost

**DOI:** 10.1371/journal.pbio.0040394

**Published:** 2006-10-24

**Authors:** Liza Gross

Males evolved extravagant plumage, towering antlers, and frenetic mating displays, Darwin proposed, because that’s what females like. Why such preferences evolved is still controversial—one view holds that flashy males beget sexy sons with more chance of attracting mates—but it’s generally thought that sexual selection provides some type of indirect genetic benefit leading to higher intrinsic fitness of the offspring. Selection on secondary sexual characteristics often results in sexually dimorphic traits being tailored toward the specific reproductive needs of each sex. Sexual dimorphism typically arises because selection operates in different directions on each gender—selecting for large males and compact females, for example—promoting sex-specific gene expression.

But when selection acts on a shared trait and the sexes are genetically constrained from becoming dimorphic, “intralocus” sexual conflict can occur. Theoretical studies predict that sexually antagonistic genes—which favor one sex to the detriment of the other—should reduce any indirect benefits of sexual selection on high-fitness parents by compromising the fitness of opposite-sex offspring. Whether this effect is short-lived, perhaps mediated by mechanisms that restrict gene expression to the favored sex, or persists as a cost of sexual reproduction is unclear.

In a new study, Alison Pischedda and Adam Chippindale explore the potential costs of intralocus sexual conflict in the genetically tractable fruit fly, *Drosophila melanogaster*. By measuring the inheritance of fitness across generations, and across the genome, they show that sexual selection provides no advantage to the next generation. To the contrary, having a fit parent of the opposite sex leads to dramatically lower rates of reproductive success. Sexually antagonistic genes, it appears, may have far-reaching effects on patterns of fitness inheritance.

Using a recently developed genetic tool called hemiclonal analysis, researchers can screen the (nearly) entire genome for genetic variation within a population and for evidence of selection acting on that variation. By manipulating chromosomal inheritance in males, hemiclonal analysis extracts, clones, and amplifies male haplotypes—single sets of the three major fruit fly chromosomes, the X chromosome, and two autosomal chromosomes—from a base population to create multiple identical haploid (single copy) genomes. These genomes, considered the functional equivalent of sperm clones, are then used to fertilize many different eggs from the original base population to create individual “hemiclones” with the same haplotype expressed against a random genetic background. With this approach, it’s possible to measure additive genetic variation in both female and male offspring and to estimate any selection acting on this variation, manifested as different fitness levels.

**Figure:**
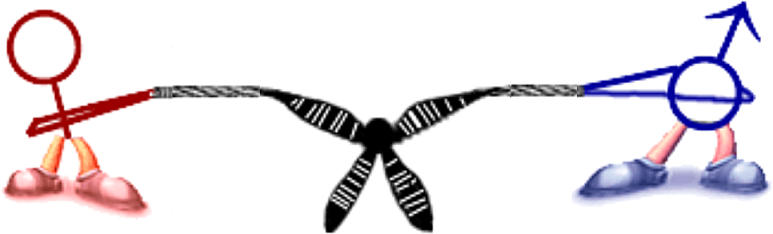
Sexually antagonistic genes create a tug-of-war over the genome. (Image: Adam Chippindale and Helene Van)

Pischedda and Chippindale used hemiclonal analysis to generate high- and low-fitness parents, and selected three lines of the most and least fit mothers and fathers, based on egg production and number of offspring sired. High-fitness females laid 35% more eggs than low-fitness females; high-fitness males fathered 44% more offspring than their less-fit counterparts. After crossing every possible combination of high- and low-fitness parental lines (yielding 36 crosses), the authors evaluated fitness effects on offspring to determine patterns of fitness inheritance, using reproductive success of sons and daughters as measures of their fitness.

Overall, they found an inverted pattern of fitness inheritance: high maternal fitness was good for daughters but not sons, and sons born of high-fitness mothers had substantially fewer offspring than those with low-fitness mothers. Similarly, daughters sired by high-fitness fathers laid fewer eggs than those with low-fitness fathers. Paternal fitness had little effect on sons’ fitness—supporting the notion that sexually antagonistic genes mostly inhabit the X chromosome, which only females transmit to sons. Thus, females that choose successful mates, the authors explain, won’t see indirect benefits through sons, and to make matters worse, will incur the cost of less-fit daughters. This sexually antagonistic pattern challenges sexual selection theory predictions that female costs of reproduction are offset by the indirect benefits of passing on good genes or generating sexy sons with high reproductive success.

Many genes shaping sexual characteristics are likely affected by the conditions that favor intralocus sexual conflict in sexually reproducing organisms, the authors argue, suggesting that the phenomenon may operate in far more organisms than the fruit fly, where it was first discovered. And because sexually antagonistic genes compromise fitness by reducing fertility, the authors suggest, they may offer clues to a longstanding puzzle: how can genetic variation for a trait persist in a population in spite of strong selection in favor of one variant? Part of the answer may lie within the X chromosome: it may harbor sexually antagonistic genes that undermine offspring fitness of one sex, despite being selected for in the other sex. For now, the assembled research suggests that sexually antagonistic genes are common and consequential in the genome and powerful enough to create a reversed inheritance of Darwinian fitness across the sexes. Simply seeking out the most attractive mate may have surprising implications for the offspring.

